# Integrative Physiological, Metabolomic and Transcriptomic Analyses Uncover the Mechanisms Underlying Differential Responses of Two *Anubias* Genotypes to Low-Temperature Stress

**DOI:** 10.3390/biom15111520

**Published:** 2025-10-28

**Authors:** Yanyu Luo, Liguo Wei, Weiguang Liu, Jiwei Chen, Jinzhong Zhang, Zhijian Yang, Shaoli Huang, Yiwei Zhou

**Affiliations:** 1 Guangzhou Academy of Agricultural and Rural Sciences, Guangzhou 510335, China; 2 Guangdong Provincial Key Laboratory of Ornamental Plant Germplasm Innovation and Utilization, Environmental Horticulture Research Institute, Guangdong Academy of Agricultural Sciences, Guangzhou 510640, China

**Keywords:** *Anubias*, cold hardiness, freezing injury, metabolome, transcriptome

## Abstract

*Anubias* (Araceae) is a globally important group of ornamental aquatic plants. However, when temperatures drop to 10 °C, most species suffer obvious frostbite from cold stress, restricting winter cultivation and broader application. This study focused on two *Anubias* genotypes with distinct cold tolerance, adopting an integrated approach combining phenotypic, physiological, metabolomic, and transcriptomic analyses to reveal the mechanisms underlying their differential cold tolerance. Under 10 °C cold stress, compared with normal temperatures, the leaves of cold-tolerant *Anubias* sp. ‘Long Leaf’ (Jian) showed no significant frostbite, while cold-sensitive *Anubias barteri* var. *nana* ‘Coin Leaf’ (Jin) had clear frost damage. Both genotypes exhibited increased leaf relative electrical conductivity, malondialdehyde (MDA) content, soluble sugar content, and activities of superoxide dismutase (SOD) and catalase (CAT); “Jian” had more notable rises in SOD/CAT activities and maintained higher levels, whereas “Jin” showed greater increases in conductivity, MDA, and soluble sugar. Metabolomic and transcriptomic analyses revealed “Jian” specifically upregulated metabolites in pathways like flavone and flavonol biosynthesis and tryptophan metabolism, as well as genes related to valine, leucine, isoleucine degradation and phenylpropanoid biosynthesis pathways. ERFs, WRKYs, NACs and other transcription factors correlated with these differentially expressed genes, suggesting potential transcriptional regulation. These results provides insights for breeding cold-tolerant *Anubias* and optimizing low-temperature cultivation.

## 1. Introduction

Low temperature is a common abiotic stress in natural environments. When exposed to cold stress, plants undergo alterations in the composition and function of their cell membrane systems [1]. They also actively accumulate osmoprotectants to enhance their osmotic adjustment capacity. For instance, increasing the levels of key osmoregulatory substances such as proline and soluble sugars helps collectively regulate cellular osmotic pressure, thereby enabling adaptation to changing environmental conditions [2]. Under stress conditions, the plant antioxidant enzyme system is also activated [3]. Enzymes such as superoxide dismutase (SOD) [4], catalase (CAT) [5,6], and ascorbate peroxidase (APX) [7] are involved in scavenging excess reactive oxygen species (ROS), mitigating oxidative damage caused by ROS.

Numerous studies have indicated that plant hormones and various metabolites play crucial roles in plant cold tolerance mechanisms [8,9,10]. Abscisic acid (ABA) is currently the most extensively studied hormone in the context of plant response to low-temperature stress. Cold stress can elevate endogenous ABA levels, thereby enhancing cold resistance [11,12,13]. Additionally, low temperature affects auxin transport, which in turn influences organogenesis and morphogenesis regulated by the accumulation and polar distribution of auxin in plant tissues [14]. Other phytohormones, including jasmonic acid (JA) [15], brassinosteroids (BRs) [16], and salicylic acid (SA) [17], also play significant roles in plant cold stress responses. Furthermore, amino acids [18], soluble sugars [19], flavonoids [20], tryptophan-related metabolites [21], and other compounds contribute importantly to cold tolerance across various plant species.

To adapt to low-temperature environments, plants activate cold tolerance mechanisms. Cold signals are perceived by membrane receptors, triggering Ca^2+^ signaling and mitogen-activated protein kinase (MAPK) cascades, which regulate downstream transcription factors and cold-responsive genes [22,23,24,25,26]. The ICE1–CBF–COR pathway plays a central role, where ICE1 induces CBF expression, leading to COR gene activation and enhanced cold tolerance [27]. Other transcription factors, including AP2/ERF [28], bHLH [29,30], MYB [31], NAC [32], and WRKY [33], also contribute by regulating genes involved in hormone signaling and protective compound synthesis.

Plants of the genus *Anubias* (Araceae) have been commercialized worldwide as highly demanded aquatic ornamentals [34]. However, due to their tropical origin and preference for warm climates, they generally exhibit poor cold tolerance [35,36]. Based on our cultivation experience, *Anubias* growth nearly ceases when temperatures fall below 15 °C, and chilling injury occurs in most varieties below 10 °C. Thus, in simple greenhouse cultivation, *Anubias* struggle to overwinter in the Guangzhou region of China without additional heating. To date, research on *Anubias* has primarily focused on plastid genomes [36], propagation [37], and disease [38,39], with no studies addressing its cold tolerance. In the course of cultivation, it was observed that two varieties, *Anubias* sp. ‘Long Leaf’ (Jian) and *Anubias barteri* var. *nana* ‘Coin Leaf’ (Jin), exhibited significant differences in performance under low winter temperatures (approximately 10 °C). Among them, “Jian” demonstrated greater cold tolerance. We speculate that the two varieties underwent distinct physiological, biochemical, and molecular changes in response to low temperature, leading to the observed divergence in cold resistance.

This study utilizes two *Anubias* varieties showing differential responses to low temperature, integrating phenotypic, physiological, metabolomic, and transcriptomic data to reveal their physiological, biochemical, and gene expression changes under cold stress. The findings provide new insights into the cold tolerance mechanisms of *Anubias*, offering important references for breeding cold-resistant varieties and promoting related industry development.

## 2. Materials and Methods

### 2.1. Plant Materials and Stress Treatment

Two varieties of *Anubias*, namely *Anubias* sp. ‘Long Leaf’ (Jian) and *Anubias barteri* var. *nana* ‘Coin Leaf’ (Jin), grown for nine months, were used as experimental materials. For cold treatment, plants of both varieties were placed in an artificial climate chamber at 10 °C for three days. Control plants were maintained at 27 ± 2 °C. Each treatment included five plants per replicate, with three biological replicates. After three days, leaf samples were collected, immediately frozen in liquid nitrogen, and stored at −80 °C for subsequent analysis. For clarity and consistency in description, the control samples of “Jian” and “Jin” under normal temperature conditions are designated as Jian-CK and Jin-CK, respectively, while the samples subjected to low-temperature stress are referred to as Jian-LT and Jin-LT.

### 2.2. Measurement of Physiological Indicators

Relative electrolyte conductivity was measured using the soaking method described by Chen et al. [40]. Activities of superoxide dismutase (SOD) and catalase (CAT), as well as contents of malondialdehyde (MDA) and soluble sugars, were determined using assay kits manufactured by Beijing Solarbio Science & Technology Co., Ltd. (Solarbio, Beijing, China), following the manufacturer’s instructions.

### 2.3. Non-Targeted Metabolomics Analysis Based on UPLC-MS/MS

#### 2.3.1. Metabolite Extraction

The LC/MS system for metabolomics analysis comprises a Waters Acquity I-Class PLUS ultra-high performance liquid chromatograph tandem with a Waters Xevo G2-XS QTof high-resolution mass spectrometer (Waters, Milford, CT, USA). The column utilized is a Waters Acquity UPLC HSS T3 column (1.8 µm, 2.1 × 100 mm). For both positive and negative ion modes, mobile phase A consists of 0.1% formic acid aqueous solution, while mobile phase B is 0.1% formic acid acetonitrile. The injection volume is set at 2 µL.

#### 2.3.2. LC-MS/MS Analysis

Primary and secondary mass spectrometry data were acquired in MSe mode using the Waters Xevo G2-XS QToF instrument controlled by MassLynx V4.2 software (Waters, Milford, USA). Each acquisition cycle included simultaneous dual-channel data collection at low collision energy (no collision energy) and high collision energy (ramped from 10 to 40 eV), with a scan rate of 0.2 s per spectrum. The electrospray ionization (ESI) parameters were set as follows: capillary voltage at 2500 V (positive mode) or −2000 V (negative mode), cone voltage at 30 V, ion source temperature at 100 °C, desolvation gas temperature at 500 °C, cone gas flow rate at 50 L/h, and desolvation gas flow rate at 800 L/h.

#### 2.3.3. Data Preprocessing, Annotation and Differential Metabolite Screening

Raw data from MassLynx V4.2 were processed using Progenesis QI software (v3.0) for peak picking, alignment, and preprocessing. Metabolites were identified by searching against the online METLIN database and a custom-built library available in Progenesis QI. Identifications were considered confident based on a threshold of a total score ≥ 40. Identified metabolites were classified and assigned to metabolic pathways using the KEGG and HMDB databases. Prior to differential analysis, data normality was assessed using the Shapiro–Wilk test, and homogeneity of variances was verified using Levene’s test. For each compound, fold changes between groups were calculated, and significance was assessed using a *t*-test (*p*-value). Orthogonal projections to latent structures–discriminant analysis (OPLS-DA) was performed using the R (v4.5.0) package “ropls” (v1.40.0), and model reliability was evaluated through 200 permutation tests. Variable importance in projection (VIP) values were derived from the OPLS-DA model via cross-validation. Differential metabolites were selected based on the following criteria: fold change (FC) > 1, *p*-value < 0.05, and VIP > 1. Enrichment significance of KEGG pathways among differential metabolites was tested using a hypergeometric distribution test.

### 2.4. RNA-Sequence Analysis

#### 2.4.1. Library Construction and Sequencing

RNA sequencing was performed on leaf samples collected from “Jian”, “Jin”, and their respective groups following low-temperature treatment. Each sample included three biological replicates. Total RNA was assessed for purity, concentration, and integrity to ensure the quality of the sequencing libraries. Libraries were constructed from 1 µg of total RNA per sample using the NEBNext^®^ Ultra™ RNA Library Prep Kit for Illumina^®^ (NEB, Ipswich, MA, USA) according to the manufacturer’s instructions. Poly-A mRNA was isolated using oligo (dT) magnetic beads and fragmented. First-strand cDNA was synthesized in NEBNext First Strand Synthesis Reaction Buffer (5×), followed by second-strand synthesis. After end repair, adapter ligation, and size selection (~240 bp) with the AMPure XP system (Beckman Coulter, Brea, CA, USA), the libraries were treated with USER Enzyme (NEB, Ipswich, MA, USA) and PCR-amplified. Final libraries were purified and quality-checked on an Agilent Bioanalyzer 2100 (Agilent Technologies, Santa Clara, CA, USA). Indexed libraries were clustered on a cBot system (Illumina, San Diego, CA, USA) and sequenced on an Illumina platform to generate paired-end reads.

#### 2.4.2. Quality Control and De Novo Assembly

Raw sequencing data were processed with custom Perl scripts to remove adapter sequences, poly-N reads, and low-quality sequences, yielding clean reads. Quality metrics including Q_20_, Q_30_, GC content, and duplication rate were calculated. De novo transcriptome assembly was performed using Trinity (v2.15.1) [41] with default parameters and a minimum k-mer coverage of 2.

#### 2.4.3. Gene Annotation and Differential Expression Analysis

Assembled transcripts were annotated against the following databases: NR (Non-Redundant Protein Sequence Database at NCBI), Pfam (Protein Family Database), KOG/COG/eggNOG (Clusters of Orthologous Groups), Swiss-Prot (Curated Protein Sequence Database), KEGG (Kyoto Encyclopedia of Genes and Genomes), and GO (Gene Ontology). Gene expression levels were estimated using RSEM (v1.3.3) by mapping clean reads to the assembled transcriptome. Differential expression analysis was conducted with the DESeq R package (v1.10.1), applying a negative binomial model. Genes with an adjusted *p*-value < 0.05 (Benjamini–Hochberg correction) were considered differentially expressed (DEGs). Functional enrichment analyses of DEGs for GO terms and KEGG pathways were performed using topGO (v2.60.1) (Kolmogorov–Smirnov test) and KOBAS (v3.0) [42,43], respectively.

### 2.5. Correlation Network Analysis

To explore associations between transcription factors (TFs) and key enzyme genes in pathways of interest, Pearson correlation analysis was conducted between differentially expressed TFs and structural genes. Significant correlations were defined as those with an absolute correlation coefficient > 0.95 and a *p*-value < 0.05. The resulting network was visualized using Cytoscape version 3.10.1 [44].

### 2.6. Statistical Analysis

Unless otherwise stated, all statistical analyses were performed in the R (v4.5.0) programming environment. One-way ANOVA, principal component analysis (PCA), and Pearson correlation analyses were carried out using built-in R functions. Hierarchical clustering heatmaps were generated with the “ComplexHeatmap” package (v2.24.1). OPLS-DA and VIP value calculations were implemented using the “ropls” package (v1.40.0).

## 3. Results

### 3.1. Effects of Low Temperature on Leaf Phenotype and Physiological Indicators

**Figure 1:**
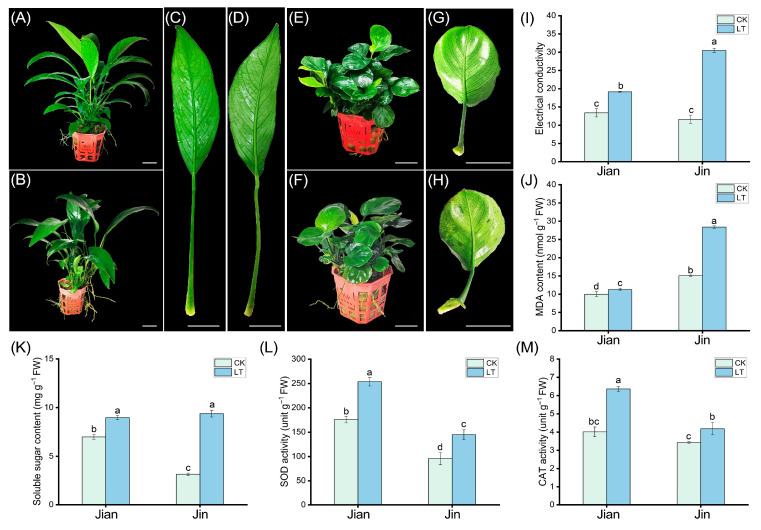
Phenotypic and physiological effects of low-temperature stress on *Anubias* sp. ‘Long Leaf’ (Jian) and *Anubias barteri* var. *nana* ‘Coin Leaf’ (Jin). (**A**,**B**) Phenotype of “Jian” plants under control and low-temperature conditions, respectively. (**C**,**D**) Leaf performance of “Jian” under control and low-temperature conditions, respectively. (**E**,**F**) Phenotype of “Jin” plants under control and low-temperature conditions, respectively. (**G**,**H**) Leaf performance of “Jin” under control and low-temperature conditions, respectively. (**I**) Relative electrolyte conductivity. (**J**) MDA content. (**K**) Soluble sugar content. (**L**) SOD activity. (**M**) CAT activity. CK: control group at ambient temperature. LT: low-temperature treatment at 10 °C. Bars with different letters (a, b, c, d) indicate significant differences (*p* < 0.05) according to one-way ANOVA.

This study investigated the impact of low temperature on two varieties of *Anubias*: the relatively cold-tolerant *Anubias* sp. ‘Long Leaf’ (Jian) and the cold-sensitive *Anubias barteri* var. *nana* ‘Coin Leaf’ (Jin). Phenotypic observations revealed no significant changes on either the adaxial or abaxial leaf surfaces of “Jian” after cold treatment. In contrast, the abaxial surface of “Jin” exhibited water-soaked lesions symptomatic of chilling injury (Figure 1). These phenotypic results indicate that “Jin” is more susceptible to cold stress than “Jian”. To further evaluate the differences in cold tolerance between the two varieties, key physiological indicators were measured. Following low-temperature stress, the relative electrolyte conductivity of “Jian” increased to 1.4 times that of the control, while that of “Jin” increased to 2.6 times. The malondialdehyde (MDA) content in “Jian” rose to 1.1 times the control level, whereas in “Jin”, it increased to 1.8 times. These results suggest more severe membrane damage in “Jin”. The antioxidant enzymes superoxide dismutase (SOD) and catalase (CAT) help alleviate oxidative damage caused by reactive oxygen species (ROS) accumulation under cold stress. Both varieties showed increased SOD and CAT activities after cold treatment. Specifically, the SOD activity in “Jian” increased to 1.4 times that of the control, compared to 1.5 times in “Jin”. The CAT activity increased to 1.5 times the control in “Jian” and 1.2 times in “Jin”. Additionally, soluble sugar content increased in both varieties under cold stress. “Jian” exhibited a 1.3-fold increase compared to the control, while “Jin” showed nearly a 3-fold increase.

### 3.2. Non-Targeted Metabolite Detection, Identification, and Annotation

**Figure 2:**
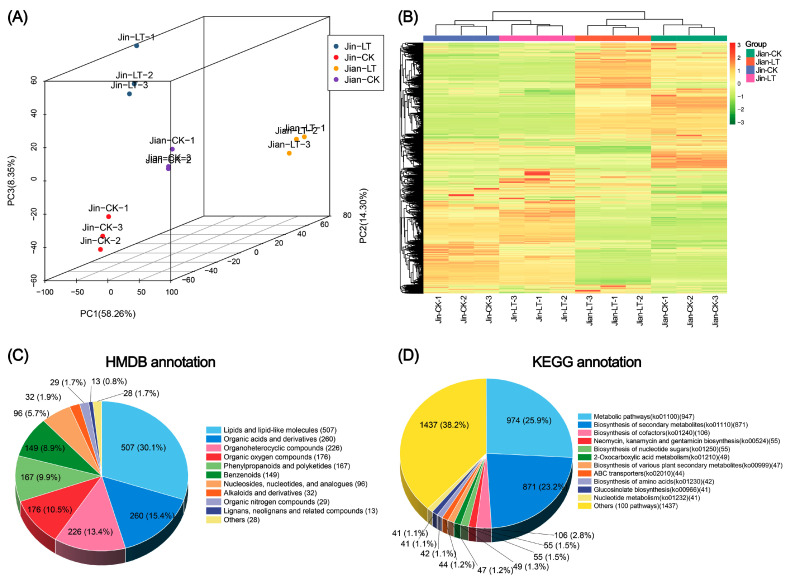
Non-targeted metabolite profiling and annotation based on UPLC-MS/MS in two *Anubias* varieties under control and low-temperature stress conditions. (**A**) PCA score plot. (**B**) Hierarchical clustering heatmap. (**C**) HMDB database annotation of metabolites. (**D**) KEGG database annotation of metabolites.

To further investigate metabolic changes in the leaves of the two *Anubias* varieties under low-temperature conditions, non-targeted metabolomic analysis was performed using UPLC-MS/MS. As shown in Figure 2, distinct metabolic differences were observed between the two species under both normal and low-temperature conditions. Principal component analysis (PCA) and hierarchical clustering analysis (HCA) revealed that the three biological replicates within each treatment group clustered closely together, indicating high data quality.

A total of 3366 metabolites were identified, including 2159 in positive ion mode and 1207 in negative ion mode (Table S1). Metabolite annotation results showed that 1678 metabolites were annotated in the HMDB database, with more than 100 metabolites each in the following categories (Table S2): Lipids and lipid-like molecules (508), organic acids and derivatives (262), organoheterocyclic compounds (227), organic oxygen compounds (177), phenylpropanoids and polyketides (167), and benzenoids (149). In the KEGG database, 2023 compounds were annotated and enriched in 112 metabolic pathways (Table S2). The pathways with the highest number of annotated metabolites were “metabolic pathways” (947, 25.9%), “biosynthesis of secondary metabolites” (871, 23.2%) and “biosynthesis of cofactors” (106, 2.8%). The number of compounds assigned to all other pathways was fewer than 100.

### 3.3. Metabolic Changes Under Low-Temperature Stress

**Figure 3:**
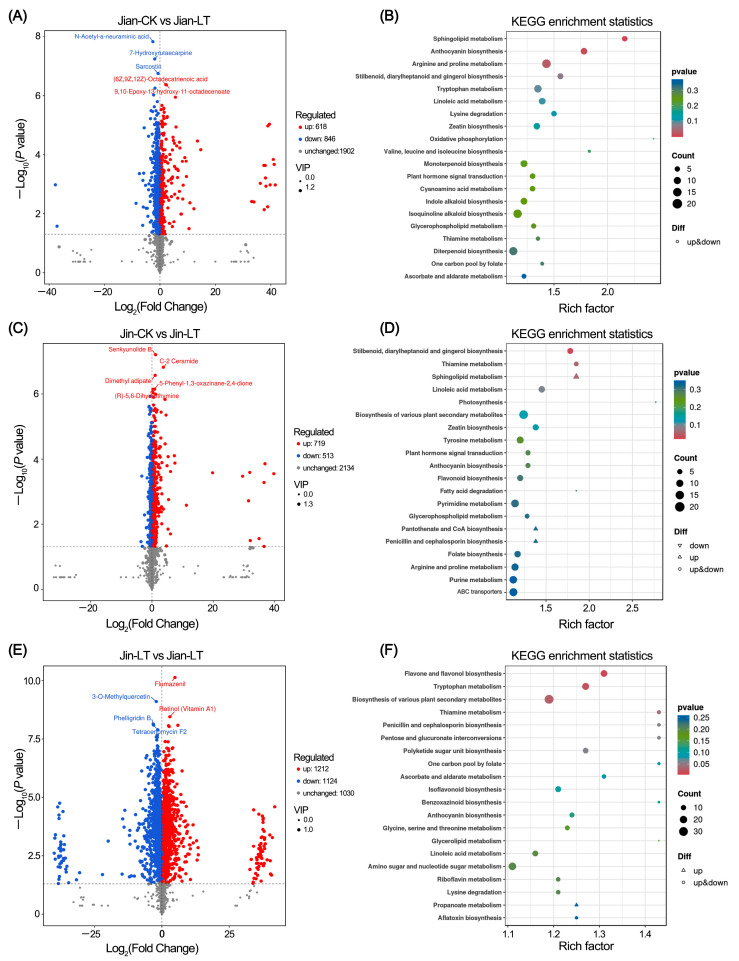
Identification of differential metabolites in two *Anubias* varieties under control versus low-temperature stress. (**A**,**B**) Volcano plot and KEGG enrichment analysis of differential metabolites in “Jian” under control and low-temperature stress, respectively. (**C**,**D**) Volcano plot and KEGG enrichment analysis of differential metabolites in “Jin” under control and low-temperature stress, respectively. (**E**,**F**) Volcano plot and KEGG enrichment analysis of differential metabolites in “Jian” versus “Jin” under low-temperature stress.

Further analysis identified changes in leaf metabolites under low-temperature conditions in both varieties (Figure 3). In the cold-tolerant variety “Jian”, 618 metabolites were up-regulated and 846 were down-regulated compared to the control conditions. In the cold-sensitive variety “Jin”, 719 metabolites were up-regulated and 513 were down-regulated under cold stress. KEGG enrichment analysis revealed that the differentially expressed metabolites in “Jian” were significantly enriched in sphingolipid metabolism, anthocyanin biosynthesis, and arginine and proline metabolism pathways. In contrast, the differential metabolites in “Jin” were primarily enriched in stilbenoid, diarylheptanoid and gingerol biosynthesis pathways. Under low-temperature treatment, compared to the cold-sensitive “Jin”, the cold-tolerant “Jian” exhibited 1212 up-regulated and 1124 down-regulated metabolites. These were significantly enriched in flavone and flavonol biosynthesis, tryptophan metabolism, biosynthesis of various plant secondary metabolites, and thiamine metabolism pathways.

**Figure 4:**
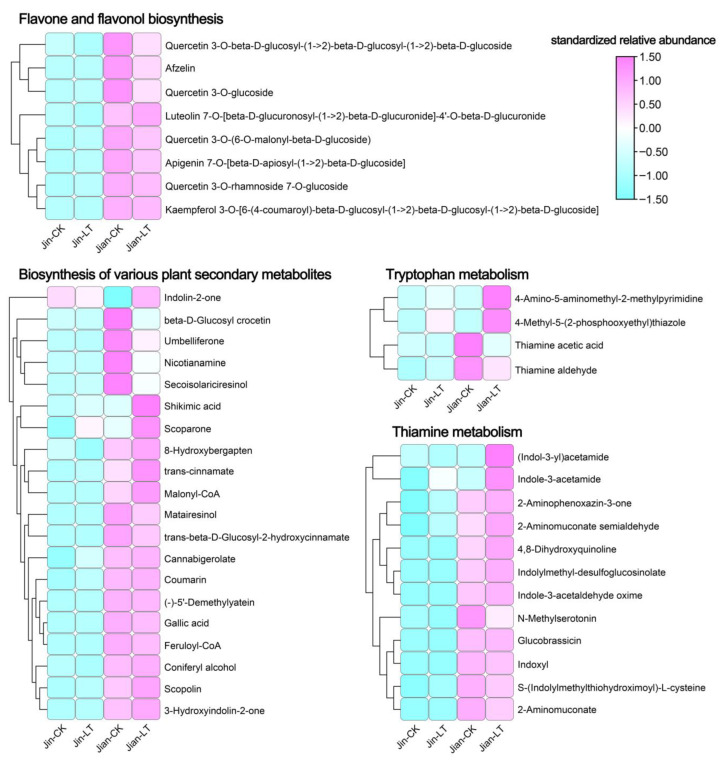
Identification of significantly up-regulated metabolites in key enriched pathways of differential metabolites in Jin-LT versus Jian-LT under low-temperature stress.

We focused specifically on metabolites in these significantly enriched pathways under low-temperature conditions (Figure 4). Compared to “Jin”, the cold-tolerant “Jian” showed 8, 12, 20, and 4 significantly up-regulated metabolites in the flavone and flavonol biosynthesis, tryptophan metabolism, biosynthesis of various plant secondary metabolites, and thiamine metabolism pathways, respectively, highlighting their potential role in cold resistance.

### 3.4. Transcriptomic Analysis Under Low-Temperature Stress

#### 3.4.1. RNA-Seq Quality Assessment, Assembly, and Annotation

To further elucidate the molecular regulatory mechanisms underlying the differential cold tolerance between the two varieties under low-temperature stress, a de novo RNA-seq analysis was conducted. A total of 265,898,237 reads (79.46 Gb) were obtained from the four samples. The GC content ranged from 48.91% to 51.40%, Q_20_ from 97.11% to 97.51%, and Q_30_ from 92.28% to 93.16%, indicating high-quality sequencing data (Table S3). After de novo assembly and gene annotation, 99,874 unigenes were annotated across nine databases: COG, GO, KEGG, KOG, Pfam, SwissProt, TrEMBL, eggNOG, and NR, with 6313, 25,196, 18,982, 15,879, 17,359, 17,003, 31,342, 25,211, and 30,296 genes annotated in each database, respectively (Table S4). In total, 32,900 genes were functionally annotated.

The box-plot demonstrates highly consistent distributions of log_10_(FPKM) across all samples, indicating robust and reproducible transcriptomic data quality (Figure S1). PCA of gene expression levels showed strong correlations among biological replicates of the same sample, which clustered closely together in the PCA score plot (Figure S2). In contrast, different samples exhibited lower correlations and greater spatial separation, confirming good reproducibility of the RNA-seq data and significant transcriptional differences between samples.

#### 3.4.2. Identification of Differentially Expressed Genes (DEGs)

**Figure 5:**
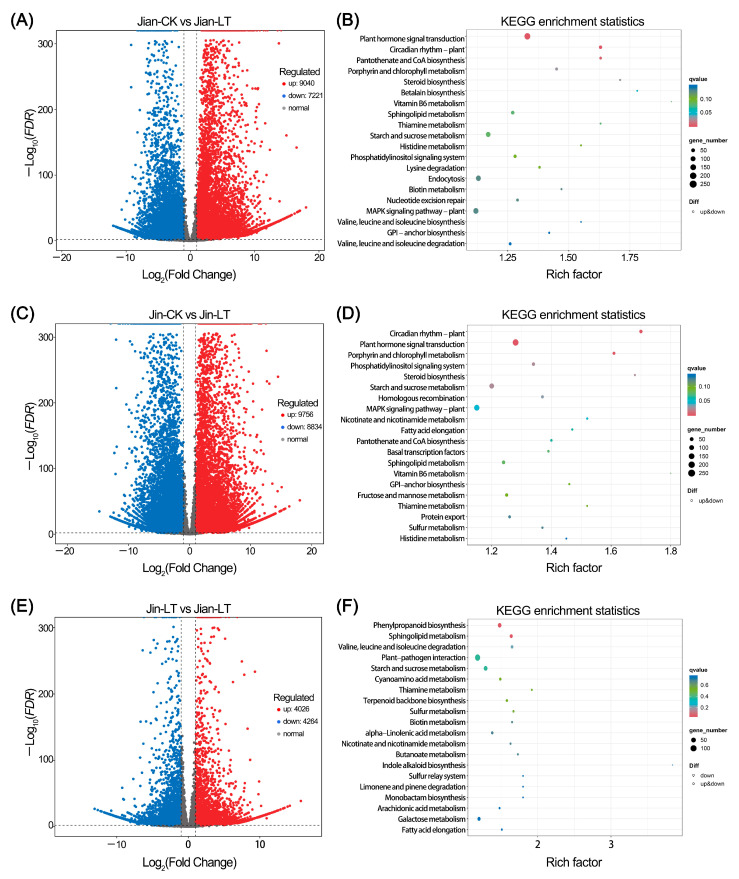
Mining of differentially expressed genes (DEGs) in two *Anubias* varieties under control and low-temperature stress. (**A**,**B**) Volcano plot and KEGG enrichment analysis of DEGs in “Jian” under control and low-temperature stress, respectively. (**C**,**D**) Volcano plot and KEGG enrichment analysis of DEGs in “Jin” under control and low-temperature stress, respectively. (**E**,**F**) Volcano plot and KEGG enrichment analysis of DEGs in “Jian” versus “Jin” under low-temperature stress.

The results of differential gene expression analysis are shown in Figure 5. Compared to the control, 9040 genes were up-regulated and 7221 were down-regulated in the cold-treated “Jian”. These DEGs were significantly enriched in pathways including Plant hormone signal transduction, Circadian rhythm-plant, Pantothenate and CoA biosynthesis, Porphyrin metabolism, and Steroid biosynthesis. Up-regulated DEGs were notably enriched in the MAPK signaling pathway-plant (122 genes), sphingolipid metabolism (51 genes), endocytosis (107 genes), plant hormone signal transduction (126 genes), valine, leucine and isoleucine degradation (31 genes), phagosome (50 genes), cysteine and methionine metabolism (63 genes), and proteasome (29 genes) (Table S5). Down-regulated DEGs were significantly enriched in circadian rhythm-plant (50 genes), porphyrin metabolism (37 genes), steroid biosynthesis (18 genes), pantothenate and CoA biosynthesis (27 genes), glycosylphosphatidylinositol (GPI)-anchor biosynthesis (16 genes), histidine metabolism (14 genes), plant hormone signal transduction (128 genes), and valine, leucine and isoleucine biosynthesis (11 genes) (Table S5).

In the cold-treated “Jin”, 9756 genes were up-regulated and 8834 were down-regulated. These were significantly enriched in pathways such as circadian rhythm-plant, plant hormone signal transduction, porphyrin metabolism, starch and sucrose metabolism, steroid biosynthesis, phosphatidylinositol signaling system, homologous recombination, nicotinate and nicotinamide metabolism, and MAPK signaling pathway-plant. Up-regulated DEGs were enriched in MAPK signaling pathway-plant (126 genes), sphingolipid metabolism (52 genes), plant hormone signal transduction (126 genes), cysteine and methionine metabolism (67 genes), endocytosis (93 genes), biosynthesis of amino acids (108 genes), proteasome (28 genes), protein processing in endoplasmic reticulum (127 genes), and phagosome (47 genes) (Table S6). Down-regulated DEGs were enriched in circadian rhythm-plant (57 genes), porphyrin metabolism (45 genes), steroid biosynthesis (18 genes), fatty acid biosynthesis (35 genes), GPI-anchor biosynthesis (19 genes), fatty acid elongation (21 genes), homologous recombination (35 genes), and basal transcription factors (25 genes) (Table S6).

Compared to the cold-sensitive “Jin”, the cold-tolerant “Jian” under low-temperature treatment exhibited 4026 up-regulated and 4264 down-regulated genes. These were mainly enriched in phenylpropanoid biosynthesis, sphingolipid metabolism, valine, leucine and isoleucine degradation, and Starch and sucrose metabolism. Up-regulated DEGs were significantly enriched in valine, leucine and isoleucine degradation (23 genes) and phenylpropanoid biosynthesis (42 genes), while down-regulated DEGs showed no significant pathway enrichment (Table S7).

**Figure 6:**
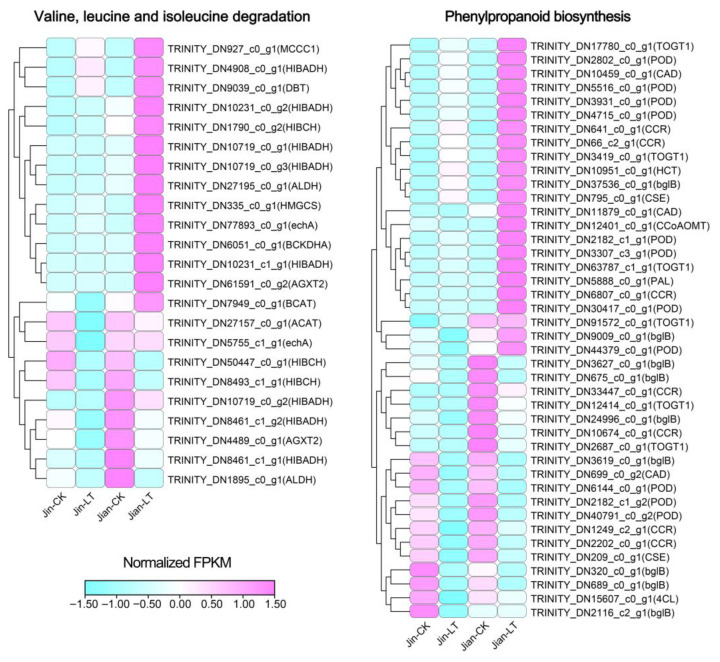
Relative expression analysis of genes related to the valine, leucine and isoleucine degradation and phenylpropanoid biosynthesis pathways, which were significantly enriched among up-regulated DEGs in Jin-LT versus Jian-LT.

We further identified key genes in the significantly enriched pathways among up-regulated DEGs in Jian-LT vs. Jin-LT (Figure 6; Table S8). In valine, leucine and isoleucine degradation, 23 genes were identified, including 8 *HIBADH*, 3 *HIBCH*, 2 *AGXT2*, 2 *ALDH*, 2 *echA*, and one each of *ACAT*, *BCAT*, *BCKDHA*, *DBT*, *HMGCS*, and *MCCC1*. The sustained high expression of these genes in “Jian” under cold stress suggests their potential role in maintaining amino acid homeostasis and providing energy for the respiratory chain, thereby contributing to cold tolerance. In phenylpropanoid biosynthesis, 42 genes were identified, including 11 *POD*, 9 *bglB*, 7 *CCR*, 6 *TOGT1*, 3 *CAD*, 2 *CSE*, and one each of *PAL*, *4CL*, *HCT*, and *CCoAOMT*. The relatively high expression of these genes may promote the synthesis of downstream antioxidants such as phenylpropanoids and flavonoids, further enhancing cold resistance in “Jian”.

#### 3.4.3. Differences in Gene Numbers in Commonly Enriched Pathways of DEGs Under Control and Low-Temperature Stress

We first analyzed the differences in the number of DEGs between the two comparison groups, Jin-CK vs. Jian-CK and Jin-LT vs. Jian-LT. Among the up-regulated genes, only 624 DEGs were shared by both comparison groups, while 1,604 were unique to Jin-CK vs. Jian-CK and 2164 were unique to Jin-LT vs. Jian-LT. Among the down-regulated genes, 416 DEGs were common to both groups, whereas 1728 were specific to Jin-CK vs. Jian-CK and 1769 were specific to Jin-LT vs. Jian-LT. These results indicate that the transcriptional responses to low temperature are markedly different between “Jin” and “Jian”.

**Figure 7:**
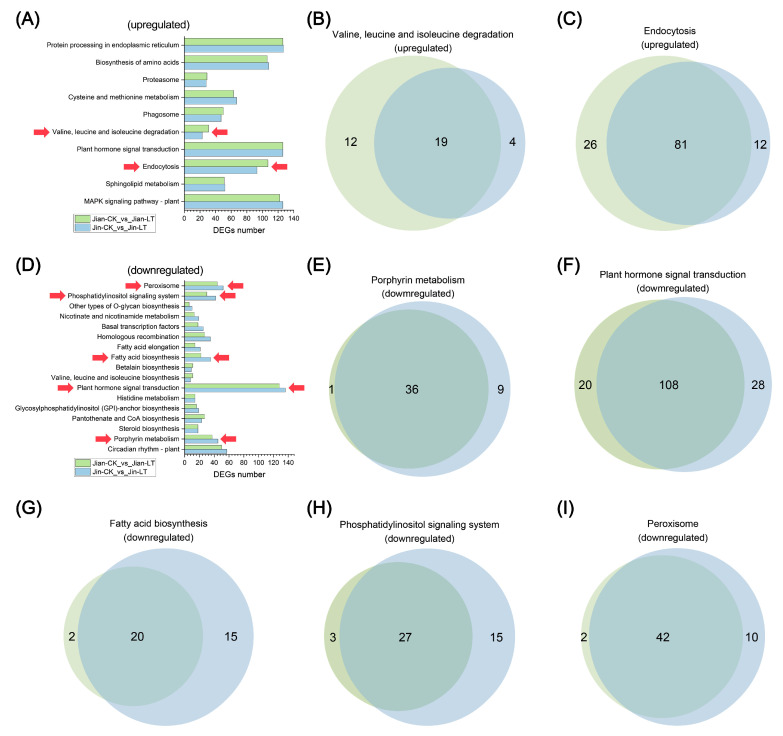
Analysis of differences in gene numbers within commonly enriched pathways of DEGs in two *Anubias* varieties under control and low-temperature stress. (**A**) Comparative analysis of the number of up-regulated DEGs. (**B**) Venn diagram of DEGs in the Valine, leucine and isoleucine degradation pathway. (**C**) Venn diagram of DEGs in the Endocytosis pathway. (**D**) Comparative analysis of the number of down-regulated DEGs. (**E**) Venn diagram of DEGs in the porphyrin metabolism pathway. (**F**) Venn diagram of DEGs in the endocytosis pathway. (**G**) Venn diagram of DEGs in the fatty acid biosynthesis pathway. (**H**) Venn diagram of DEGs in the phosphatidylinositol signaling system pathway. (**I**) Venn diagram of DEGs in the peroxisome pathway.

To further investigate key metabolic pathways altered by low-temperature stress in both varieties, we compared the number of DEGs in significantly enriched pathways under cold conditions (Figure 7). Among the significantly up-regulated pathways, notable differences in DEG numbers were observed in Valine, leucine and isoleucine degradation and Endocytosis. Only 19 and 81 DEGs were shared between the two varieties in these pathways, respectively. The cold-tolerant variety “Jian” exhibited a substantially greater number of unique up-regulated genes in these pathways compared to “Jin”. For significantly down-regulated pathways, considerable differences in DEG abundance were identified in porphyrin metabolism, plant hormone signal transduction, fatty acid biosynthesis, phosphatidylinositol signaling system, and peroxisome pathways. Specifically, the cold-sensitive variety “Jin” showed markedly more unique down-regulated DEGs in these pathways, with 9, 28, 15, 15, and 10 unique genes, respectively, compared to “Jian”.

#### 3.4.4. Analysis of Differentially Expressed Transcription Factors

**Figure 8:**
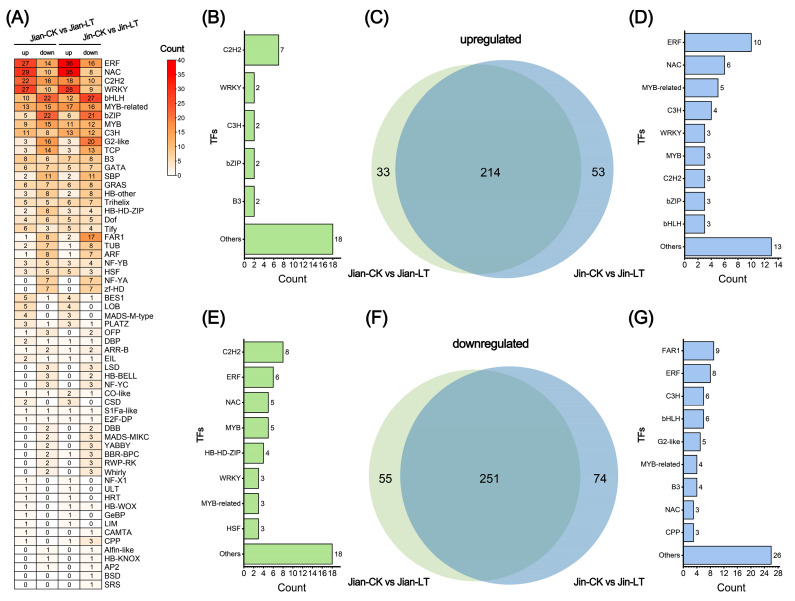
Statistical analysis of transcription factors (TFs) with significant expression changes under low-temperature stress in two *Anubias* varieties. (**A**) Number of differentially expressed TFs in “Jian” and “Jin” compared to control. (**B**) Number of TFs significantly up-regulated only in Jian-CK vs. Jian-LT. (**C**) Venn analysis of TFs significantly up-regulated in both Jian-CK vs. Jian-LT and Jin-CK vs. Jin-LT. (**D**) Number of TFs significantly up-regulated only in Jin-CK vs. Jin-LT. (**E**) Number of TFs significantly down-regulated only in Jian-CK vs. Jian-LT. (**F**) Venn analysis of TFs significantly down-regulated in both Jian-CK vs. Jian-LT and Jin-CK vs. Jin-LT. (**G**) Number of TFs significantly down-regulated only in Jin-CK vs. Jin-LT.

Differentially expressed transcription factors (TFs) were identified and statistically analyzed following low-temperature stress (Figure 8). Compared to the control, 247 TFs were up-regulated and 306 were down-regulated in cold-treated “Jian”. The TF families exhibiting the most pronounced changes (over 20 members each) included ERF, NAC, C2H2, WRKY, bHLH, MYB-related, bZIP, and MYB. Similarly, in cold-treated “Jin”, 247 TFs were up-regulated and 306 down-regulated, with substantial changes (over 20 members each) observed in ERF, NAC, C2H2, WRKY, bHLH, MYB-related, bZIP, MYB, C3H, and G2-like families. The similarity in the TF families showing extensive expression changes suggests a conserved transcriptional response to low-temperature stress in both varieties.

To further investigate varietal differences in TF expression, we examined the consistency of significantly altered TFs under cold stress. Among the significantly up-regulated TFs, 33 were unique to “Jian”, with the C2H2 family being the most abundant. In contrast, 53 were uniquely up-regulated in “Jin”, predominantly from the ERF, NAC, MYB-related, and C3H families. Among the significantly down-regulated TFs, 55 were unique to “Jian”, mainly comprising C2H2, ERF, NAC, and MYB families, while 74 were unique to “Jin”, with FAR1, ERF, C3H, bHLH, and G2-like being the most represented. Overall, the cold-sensitive variety “Jin” exhibited a greater number of significantly altered TFs in response to cold stress compared to the cold-tolerant variety “Jian”.

### 3.5. Correlation Network Analysis Between Transcription Factors and Key Differential Metabolic Pathways

**Figure 9:**
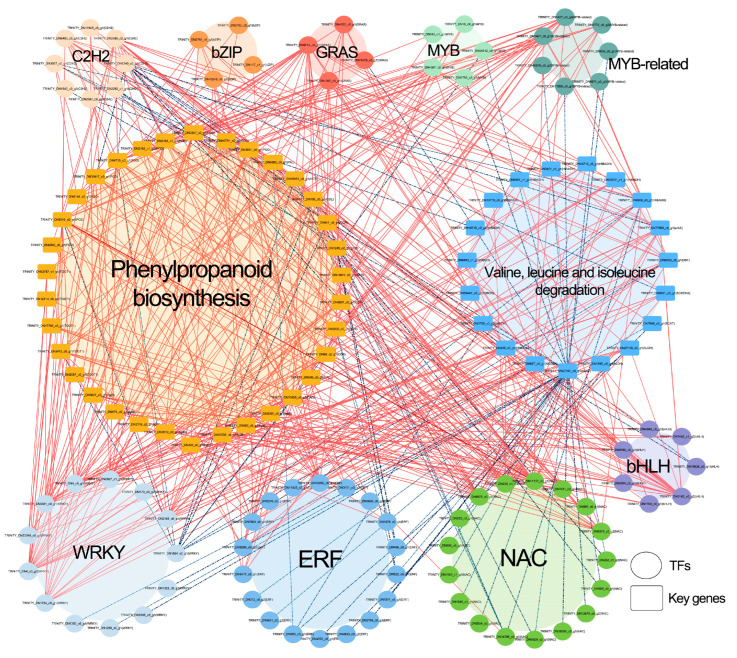
Correlation network analysis between key transcription factors (TFs) responsive to cold stress in *Anubias* and genes from significantly up-regulated metabolic pathways (Phenylpropanoid biosynthesis and Valine, leucine and isoleucine degradation). Solid red lines indicate significant positive correlations between a TF and a gene, while blue dashed lines represent significant negative correlations.

We performed correlation analysis between major reported transcription factor (TF) families—including ERF, MYB, MYB-related, bHLH, NAC, WRKY, C2H2, bZIP, and GRAS—and enzyme-coding genes from two significantly enriched pathways: Phenylpropanoid biosynthesis and Valine, leucine and isoleucine degradation (Figure 9; Table S9). The results revealed that 83 TFs, including 18 ERF, 17 NAC, 13 WRKY, 8 C2H2, 7 bHLH, 7 MYB-related, 5 MYB, 4 bZIP, and 4 GRAS members, were significantly correlated with 34 genes involved in phenylpropanoid biosynthesis and 19 genes associated with valine, leucine, and isoleucine degradation. These correlations were predominantly positive.

Specifically, 47 TFs (9 *ERF*, 8 *WRKY*, 7 *NAC*, 6 *bHLH*, and 17 others) showed significant positive correlations with 34 Phenylpropanoid biosynthesis genes (10 *POD*, 8 *bglB*, 6 *CCR*, 5 *TOGT1*, and 5 others). In contrast, 13 TFs (4 *NAC*, 3 *ERF*, 3 *C2H2*, and 3 others) were significantly negatively correlated with 16 Phenylpropanoid biosynthesis genes (6 *POD*, 5 *CCR*, and 5 others). Furthermore, 38 TFs (7 *WRKY*, 6 *ERF*, 6 *NAC*, 5 *bHLH*, and 14 others) exhibited significant positive correlations with 17 Valine, leucine and isoleucine degradation genes (6 *HIBADH*, 3 *HIBCH*, 2 *ALDH*, and 6 others). Conversely, 38 TFs (11 *NAC*, 9 *ERF*, 5 *WRKY*, 5 *C2H2*, and 8 others) were significantly negatively correlated with 11 genes in this pathway (2 *HIBADH* and 9 others). These findings highlight the potential transcriptional regulatory roles of these TFs in modulating genes involved in phenylpropanoid biosynthesis and valine, leucine and isoleucine degradation pathways.

## 4. Discussion

In this study, after three days of treatment at 10 °C, the cold-sensitive variety “Jin” exhibited clear symptoms of water-soaked chilling injury, whereas the cold-tolerant variety “Jian” did not. Although both *Anubias* varieties showed significant increases in relative electrolyte conductivity and MDA content, the increases were more pronounced in “Jin”. Additionally, soluble sugar concentration, SOD activity, and catalase CAT activity were elevated to varying degrees in both varieties. Notably, the cold-tolerant “Jian” maintained significantly higher levels of SOD and CAT activity compared to “Jin”. SOD [45] and CAT [46] play crucial roles in cold resistance by synergistically scavenging reactive oxygen species (ROS) and protecting plant Biomolecules from oxidative damage under low-temperature stress [47]. Our results clearly demonstrate that the cold-tolerant “Jian” exhibits higher antioxidant enzyme activity under cold stress than the cold-sensitive “Jin”, which is consistent with previous studies.

Numerous metabolites have been implicated in cold stress responses across various plant species. In this study, UPLC-MS/MS analysis revealed that, compared to the cold-sensitive “Jin”, the cold-tolerant “Jian” showed significant up-regulation of metabolites involved in flavone and flavonol biosynthesis, tryptophan metabolism, biosynthesis of various plant secondary metabolites, and thiamine metabolism. flavonoids, key secondary metabolites in plants, play vital roles in development and stress responses [20]. Under cold stress, plants accumulate flavonoids to provide antioxidant protection by neutralizing free radicals and ROS, thereby preventing cellular damage [48]. Flavonoid accumulation has been documented in multiple plant species under cold stress, including coconut [49], alfalfa [50], mustard [51], and barley [52]. Additionally, thiamine enhances plant immunity and defense systems, playing a key role in protection against biotic and abiotic stresses [53,54]. Cold stress has been reported to activate thiamine metabolism in *Arabidopsis* [55], grape [56], and Chinese yew [57]. Tryptophan-related metabolites also contribute significantly to stress resistance in plants [58]. Exogenous tryptophan application has been shown to enhance cold tolerance in soybean seedlings [59], and low-temperature storage modulates tryptophan metabolism to maintain the quality of high-moisture maize [60]. These findings support the important role of metabolite accumulation in flavone and flavonol biosynthesis, tryptophan metabolism, biosynthesis of various plant secondary metabolites, and thiamine metabolism in the cold tolerance of “Jian”.

Compared to control conditions, DEGs in both varieties under cold stress were significantly enriched in pathways such as plant hormone signal transduction, circadian rhythm-plant, porphyrin metabolism, steroid biosynthesis, MAPK signaling pathway-plant, sphingolipid metabolism, cysteine and methionine metabolism, proteasome, and phagosome, highlighting shared response mechanisms between the two varieties. Notably, up-regulated DEGs in the cold-tolerant “Jian” were predominantly enriched in valine, leucine and isoleucine degradation and phenylpropanoid biosynthesis compared to the cold-sensitive “Jin”. Furthermore, when compared to its own control, “Jian” under cold stress also showed significant enrichment of up-regulated DEGs in valine, leucine and isoleucine degradation, whereas “Jin” did not. Branched-chain amino acid (BCAA) catabolism not only contributes to amino acid homeostasis but also serves as an alternative energy source when carbohydrate availability is limited [61]. Under such conditions, BCAAs act as respiratory substrates, providing electrons to the respiratory chain and intermediates to the tricarboxylic acid cycle [62,63]. Studies on abiotic stresses such as drought in sugarcane [64], wheat [65], and *Hibiscus mutabilis* [66], as well as diurnal temperature variation in tea plants [67], have reported significant enrichment of DEGs in valine, leucine and isoleucine degradation, aligning with our findings and expanding the understanding of this pathway’s role in plant stress responses. The metabolic differences in this pathway between the two *Anubias* varieties under cold stress may significantly contribute to their differential cold tolerance.

**Figure 10:**
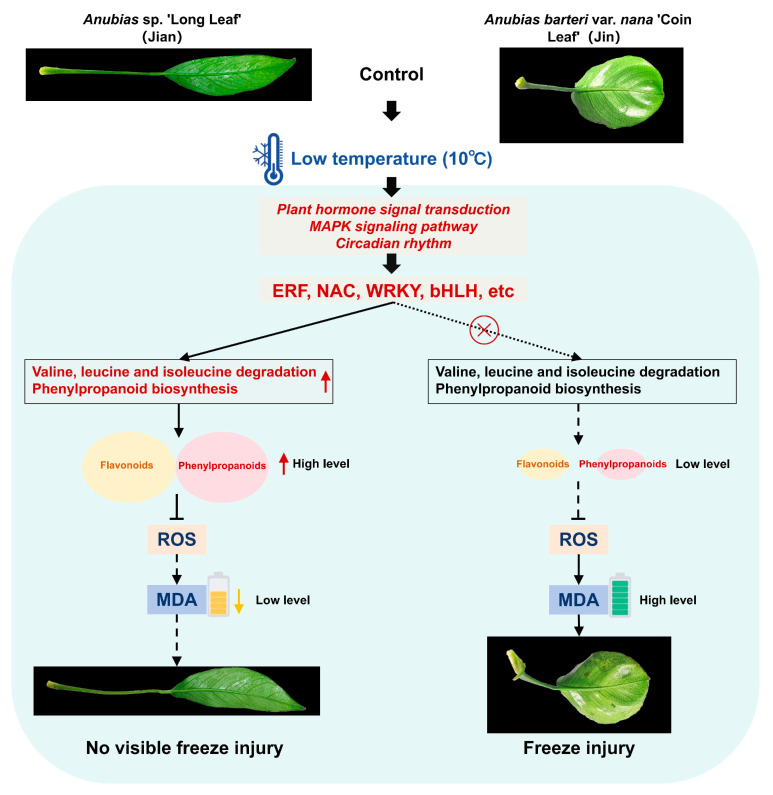
Proposed model illustrating the differential response mechanisms of two *Anubias* varieties under low-temperature stress. A solid arrow indicates a promoting effect, while a dashed arrow suggests a diminished promoting effect. A solid line with a ‘T’ end denotes inhibition, while a dashed line with a ‘T’ end suggests weaker inhibition. The red circle-with-cross symbol indicates a potentially non-existent transcriptional regulatory targeting relationship.

In recent years, multiple TFs involved in cold stress responses have been characterized [68]. TFs play essential roles in regulating growth, development, and physiological adaptation under cold stress. In this study, we identified several TFs with altered expression in both *Anubias* varieties under low-temperature conditions. Correlation analysis further suggested significant associations between specific TFs and genes in the valine, leucine and isoleucine degradation and phenylpropanoid biosynthesis pathways, implying potential regulatory relationships. We propose a model for the differential responses of the two varieties under cold stress (Figure 10). Upon exposure to 10 °C, both varieties perceive the low-temperature signal, leading to significant changes in signal transduction pathways such as plant hormone signal transduction, circadian rhythm-plant, and MAPK signaling pathway-plant, which activate various TFs (e.g., ERF, NAC, WRKY). However, unlike the cold-sensitive variety, the cold-tolerant “Jian” may employ these TFs to activate valine, leucine and isoleucine degradation—regulating amino acid homeostasis and providing energy—and to enhance phenylpropanoid biosynthesis, increasing the production of flavonoids and phenylpropanoids that scavenge ROS or reinforce cell walls to reduce oxidative damage, thereby conferring greater cold tolerance.

While this study has delineated the physiological, metabolic, and transcriptional changes in two *Anubias* varieties under low-temperature stress and highlighted the potential roles of key pathways and TFs, the functions of these key candidates—particularly the pivotal TFs—require further validation. This should include dynamic RT-qPCR analyses following low-temperature treatment, as well as genetic transformation experiments (e.g., gene overexpression and silencing) to confirm their roles in cold tolerance. In addition, targeted metabolomics should be applied to accurately quantify metabolites in key pathways implicated in the cold response—such as flavone and flavonol biosynthesis, tryptophan metabolism, biosynthesis of various plant secondary metabolites, and thiamine metabolism—to identify signature metabolites critical for cold tolerance in *Anubias*. These follow-up studies will provide a foundation for the precise evaluation and targeted breeding of cold-hardy *Anubias* varieties.

Although we have preliminarily identified key metabolic pathways contributing to the differential cold tolerance between the two varieties, the central signaling components and key regulators require further experimental validation. Additionally, the regulatory roles of the identified TFs in activating Valine, leucine and isoleucine degradation and Phenylpropanoid biosynthesis pathways need to be verified through molecular biological approaches.

## 5. Conclusions

This multi-omics investigation reveals that the differential cold tolerance between the two *Anubias* genotypes stems from distinct molecular and physiological responses to low-temperature stress. The cold-tolerant genotype *Anubias* sp. ‘Long Leaf’ (Jian) exhibited superior performance through maintaining significantly higher activities of key antioxidant enzymes (SOD and CAT), thereby mitigating oxidative damage more effectively than the cold-sensitive genotype *Anubias barteri* var. *nana* ‘Coin Leaf’ (Jin). Furthermore, “Jian” specifically activated biosynthesis of protective metabolites—particularly flavones and flavonols—and enhanced tryptophan metabolism, alongside coordinated upregulation of genes involved in phenylpropanoid biosynthesis and valine, leucine, and isoleucine degradation. Critical transcription factor families, including ERFs, WRKYs, and NACs, were implicated in regulating these adaptive responses. These findings provide a comprehensive molecular framework for understanding cold tolerance mechanisms in aquatic plants and offer valuable insights for the targeted breeding of cold-hardy *Anubias* varieties.

## Data Availability

The original contributions presented in this study are included in the supplementary material. Further inquiries can be directed to the corresponding author(s). The raw RNA-seq data have been deposited in the NCBI Sequence Read Archive (SRA) under the accession number PRJNA1348912.

## Supplementary Materials

The following supporting information can be downloaded at: https://www.mdpi.com/article/10.3390/biom15111520/s1. Table S1: UPLC-MS/MS-based non-targeted metabolite profiling of two *Anubias* cultivars under cold stress; Table S2. HMDB and KEGG database annotation of metabolites; Table S3: Summary of RNA-seq data statistics for the 12 samples; Table S4: Gene annotation statistics based on nine databases; Table S5: KEGG enrichment analysis of up- and down-regulated DEGs in Jian-CK_vs_Jian-LT; Table S6: KEGG enrichment analysis of up- and down-regulated DEGs in Jin-CK vs. Jin-LT; Table S7: KEGG enrichment analysis of up- and down-regulated DEGs in Jin-LT vs. Jian-LT; Table S8: DEG information for two significantly enriched KEGG pathways in Jin-LT vs. Jian-LT; Table S9: Significantly correlated TFs and key genes involved in phenylpropanoid biosynthesis and valine, leucine, and isoleucine degradation; Figure S1: Box-plot comparison of Log_10_(FPKM) values across 12 RNA-seq samples; Figure S2: PCA score plot evaluating the 12 RNA-seq samples; Figure S3. Venn diagram of upregulated DEGs between “Jin-CK vs. Jian-CK” and “Jin-LT vs. Jian-LT;” Figure S4. Venn diagram of downregulated DEGs between “Jin-CK vs. Jian-CK” and “Jin-LT vs. Jian-LT”.
