# Supplementary material for: Integrative Physiological, Metabolomic and Transcriptomic Analyses Uncover the Mechanisms Underlying Differential Responses of Two Anubias Genotypes to Low-Temperature Stress
Source: Biomolecules. 2025 Oct 28;15(11):1520. doi: 10.3390/biom15111520 (PMC12650335; doi:10.3390/biom15111520)
Supplement: Supplementary file 1 [file biomolecules-15-01520-s001.zip › supplementary files/Figure S1-S4.pdf]

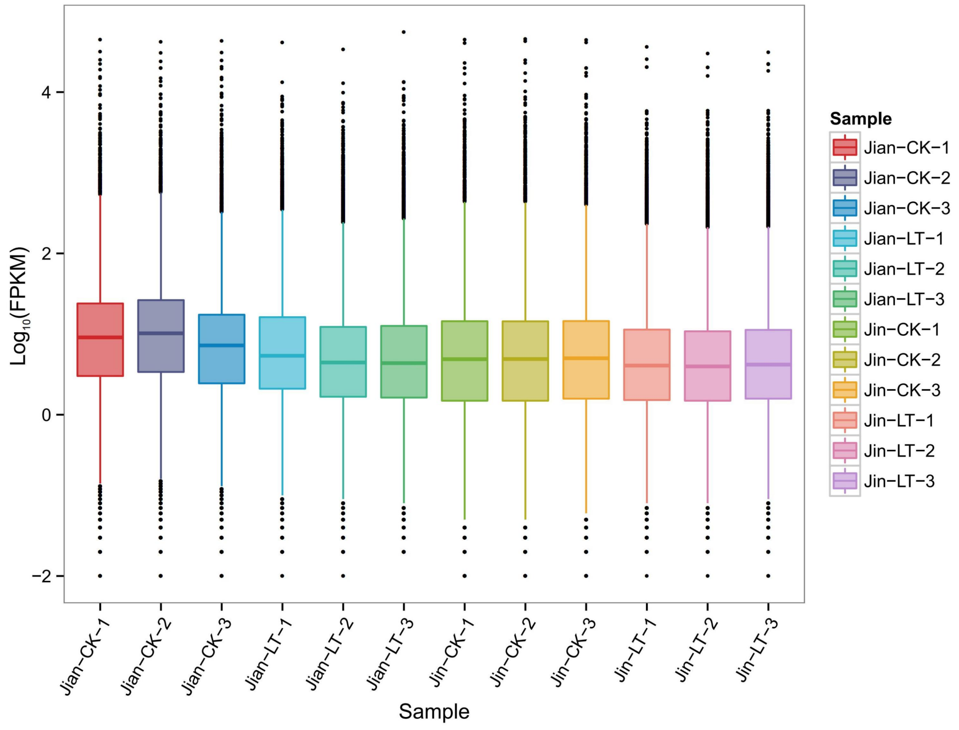

Figure S1. Box-plot comparison of Log_{10}(FPKM) values across 12 RNA-seq samples.

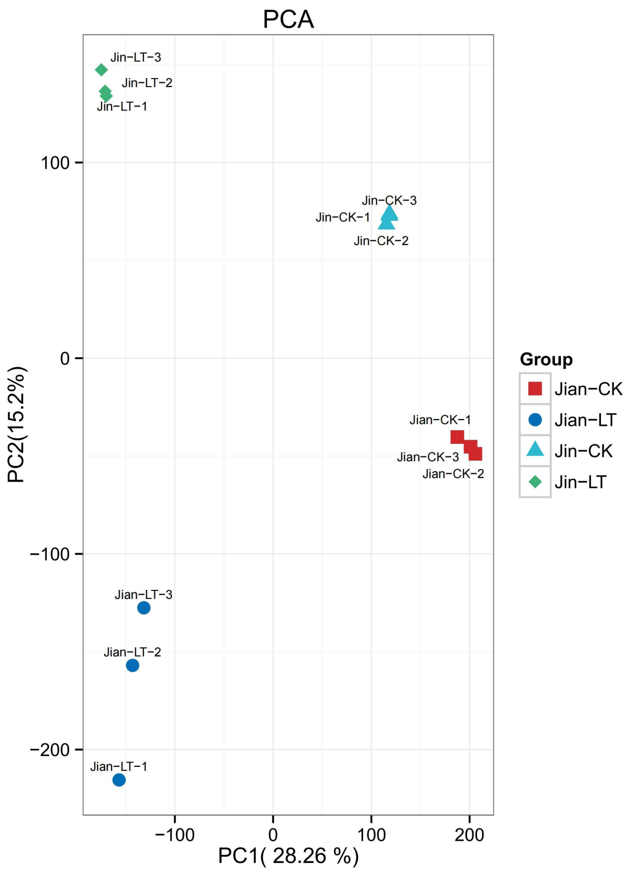

Figure S2. PCA score plot evaluating the 12 RNA-seq samples.

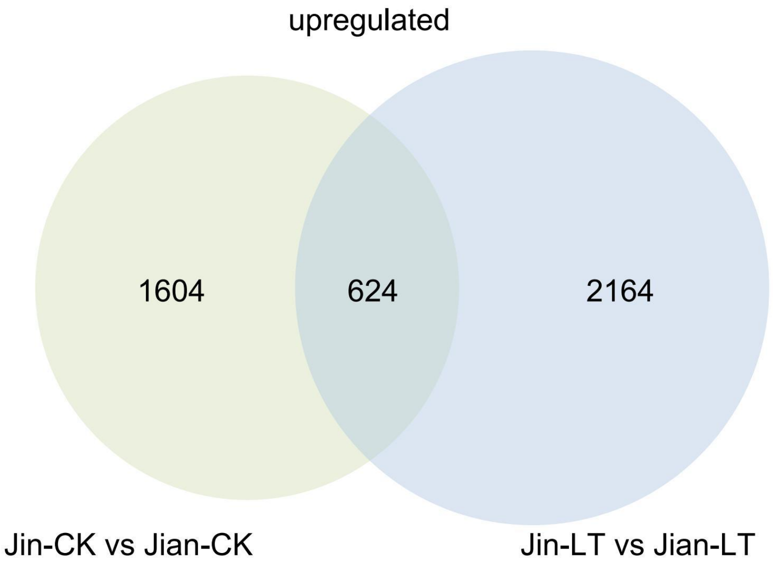

Figure S3. Venn diagram of upregulated DEGs between “Jin-CK vs Jian-CK” and “Jin-LT vs Jian-LT.”

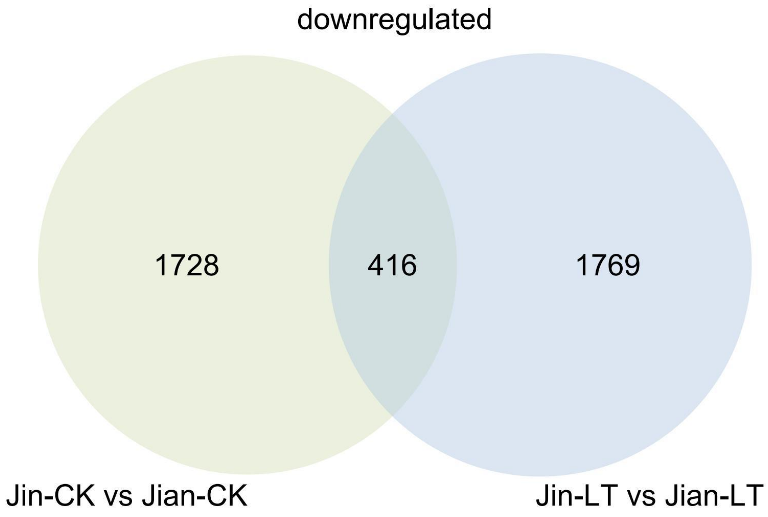

Figure S4. Venn diagram of downregulated DEGs between “Jin-CK vs Jian-CK” and “Jin-LT vs Jian-LT.”
